# Can unstimulated whole salivary flow objectively classify salivary gland secretory function in Sjögren’s syndrome?

**DOI:** 10.1007/s10067-024-07132-x

**Published:** 2024-10-07

**Authors:** Li-Qin Peng, Xing-Huan Chen, Wen-Jing Yang, Wen-Ke Huang, Zhi-Ming Ouyang, Li-Yuan Cai, Lie Dai, Ying-Qian Mo

**Affiliations:** 1 Department of Rheumatology and Immunology, Sun Yat-Sen Memorial Hospital, Sun Yat-Sen University, Guangzhou, China; 2 Department of Rheumatology and Immunology, Shenshan Medical Center, Memorial Hospital of Sun Yat-Sen University, Shanwei, China

**Keywords:** Sjögren’s syndrome, Unstimulated whole salivary flow, Xerostomia

## Abstract

**Introduction:**

The aim of this study is to investigate whether the testing time for unstimulated whole salivary flow (UWSF) can be shortened to 5 min in patients with suspected Sjögren’s syndrome (SjS); and which SjS patients can use UWSF to evaluate salivary gland (SG) secretory function.

**Method:**

A diagnostic cohort comprising suspected SjS patients was conducted to investigate the correlation between UWSF measurements taken at 10 min (UWSF_10 min) and those taken at 5 min (UWSF_5 min). A group of SjS patients was enrolled for a comparison between UWSF and stimulated whole salivary flow (SWSF).

**Results:**

In 734 suspected SjS patients, there was a remarkably high concordance between UWSF_10 min and UWSF_5 min (*ICC* 0.970, *P* < 0.001; *r* 0.973, *P* < 0.001). Reducing the testing time for UWSF to 5 min resulted in a high PPV of 83.8% and an exceptionally high NPV of 98.7%. In 408 SjS patients, the cut-off values of UWSF_10 min were investigated to classify SG secretory function. Using a threshold of > 0.2 mL/min (36.8%, 150/408) instead of SWSF > 0.7 mL/min (indicating mild secretory hypofunction), the specificity and PPV were found to be 94.2% and 94.0%, respectively; and using a threshold of < 0.05 mL/min (16.9%, 69/408) instead of SWSF ≤ 0.7 mL/min (indicating moderate to severe secretory hypofunction), the specificity was remarkably high at 97.6%, accompanied by a high PPV of 91.3%.

**Conclusions:**

This study supports the possibility of reducing UWSF testing time to 5 min; and the SWSF test may be skipped for SjS patients with USWF > 0.2 mL/min, indicating mild secretory hypofunction, or < 0.05 mL/min, indicating moderate to severe secretory hypofunction.

**Table:** 

**Key Points** •*A diagnostic cohort of 734 patients with clinical suspicion of SjS provides compelling evidence for the potential to reduce the testing time for UWSF from 10 to 5 min.* •*Our finding challenges the 2019 treatment recommendation for SjS, which does not require SWSF measurement in SjS patients with UWSF ≥ 0.1 mL/min.* •*We propose that it may be feasible to consider utilizing UWSF instead of SWSF test for objective classification of SG secretory function in over half of SjS patients.*

## Introduction

Sjögren’s syndrome (SjS) is a systemic autoimmune disease with a reported global prevalence of 3–11 cases per 100,000 people [1–4]. The disease mainly affects exocrine glands such as salivary glands (SG) and lacrimal glands, as well as various tissues and organ systems including hemocytes, lungs, renal tubules, muscles, and nerves. Of newly diagnosed SjS patients 90% have xerostomia as a symptom [2, 5], indicating that SG were involved the most.

Subjective xerostomia is not a reliable indicator for evaluating SG secretory function. It is considered as part of eligibility determination rather than being used as criteria in the new classification criteria for SjS [6]. Unstimulated whole salivary flow (UWSF) less than or equal to 0.1 mL/min objectively indicates xerostomia in different SjS classification criteria [6, 7]. The collection time of UWSF measurement was 15 min in the classical 2002 AECG classification criteria [7]. However, the 15-min duration of UWSF testing is considered time-consuming. Some clinicians believe that UWSF is only performed when objective measurements for dry eye, such as the Schirmer test or ocular staining score, yield negative results [6]. The collection time is attempting to be shortened and therefore nearly all clinical application of UWSF measurement prefers a period of 10 min, not only in clinical trials but also in clinical practice [8, 9]. The recent 2016 ACR/EULAR criteria no longer constrain the collection time [6]. Other studies are further attempting to shorten to 5 min [10, 11]. Up to date, none of studies questioned whether UWSF measurement can be shorten from 10 to 5 min in patients with suspected SjS.

The SG secretory function is measured by UWSF and stimulated whole salivary flows (SWSF). According to European League Against Rheumatism (EULAR) recommendations for the SjS management in 2019, the preferred first therapeutic approach for oral dryness based on the SG secretory function [12]. SWSF should be measured in SjS patients with UWSF < 0.1 mL/min. SjS patients can be stratified into mild hypofunction (SWSF > 0.7 mL/min) who are recommended non-pharmacological stimulation; moderate hypofunction (SWSF 0.1 ~ 0.7 mL/min) who are recommended pharmacological stimulation, and severe hypofunction (SWSF < 0.1 mL/min) who are recommended saliva substitution. However, SWSF is relatively complex in daily practice and not suitable for use in all clinical settings [13]. To our knowledge, no study was interested in whether UWSF which has been already measured when diagnosing SjS can play a similar role to SWSF in stratifying SG secretory function.

This study aimed at (i) identifying whether UWSF measurement can be shorten from 10 to 5 min in patients with suspected SjS; and (ii) identifying whether UWSF measurement can stratify SG secretory function in parts of SjS patients.

## Materials and methods

### Study design and participants

This is a cross-sectional study. The suspected SjS patients presented with ocular dryness and/or oral dryness based on American-European Consensus Group (AECG) questions [14] were recruited from September 2021 to October 2023 both in Sun Yat-Sen Memorial Hospital (Guangzhou, China) and Shenshan Medical Center of Sun Yat-Sen Memorial Hospital (Shanwei, China). Patients were excluded if they were classified as SjS overlapped with systemic lupus erythematosus, rheumatoid arthritis, or other connective tissue diseases (CTD); or if they were unable to receive the designated clinical measurements; or if they had a history of head and neck radiation treatment, active hepatitis C infection, acquired immune deficiency syndrome, sarcoidosis, amyloidosis, graft-versus-host disease, or immunoglobulin (Ig)G4-related disease. Participants meeting the 2016 American College of Rheumatology (ACR)/EULAR classification criteria for SjS [6] were classified as SjS patients, while those not meeting the criteria were classified as non-SjS controls. The protocol was approved by the Medical Ethics Committee of Sun Yat-sen Memorial Hospital (SYSEC-KY-KS-2018–012) and the Medical Ethics Committee of Shenshan Medical Center (2023-SSKY-575). All participants provided written informed consent forms. This study was conducted in compliance with the Helsinki Declaration.

### Clinical measurement

Demographic and clinical data were collected at enrollment including sex, age, ocular signs, laboratory examination, labial SG biopsy, extra-glandular involvements, etc. SjS patients were additionally assessed EULAR Sjögren’s Syndrome Disease Activity Index (ESSDAI) [15], and EULAR Sjögren’s Syndrome Patient Indexes (ESSPRI) [16].

### Total salivary flow rate measurements

Participants who are regularly taking anticholinergic medications should be evaluated after a sufficient period of time off these medications. All participants received UWSF measurement in a noise-free setting, refraining from any water intake for at least 30 min prior to the measurement. To successively get results of UWSF_5 min and UWSF_10 min, we prepared two containers for each participant. UWSF measurement was carried out over a period of 10 min using standard methods [11]. Saliva of the initial five minutes which was spilt into one container was measured and divided by five to get the milliliters per minute as UWSF_5 min result. Saliva of the latter 5 min was spilt into the other container. The saliva volume from the initial 5 min to the ultimate five minutes was divided by ten to get the milliliters per minute as UWSF_10 min result. UWSF ≤ 0.1 mL/min supporting the diagnosis of SjS was considered as positive UWSF [6, 7].

Just after UWSF measurement, SjS patients additionally received SWSF measurement which is required in clinical routine. SWSF using chewing glucose-free gum at a rate of 60–70 times per minute [11]. Accordingly, SG secretory hypofunction was stratified into mild (SWSF > 0.7 mL/min), moderate (SWSF 0.1 ~ 0.7 mL/min), and severe (SWSF < 0.1 mL/min).

### Online questionnaire survey

A large-scale questionnaire survey was conducted using an online survey tool, Sojump Survey Software (Shanghai Information Co., Shanghai, China), to evaluate SG secretory function across multiple centers all over China between May and July 2023. It was carried out with the participation of the Sjogren's Syndrome Group of the Rheumatologists and Immunologists Branch of the Chinese Medical Doctors’ Association. The survey invited experienced Chinese clinicians in diagnosing and treating SjS. The clinicians voluntarily and independently completed the questionnaire without any conflict of interest. The questionnaire focused on the prevalence of SWSF measurement and the challenges associated with its implementation.

### Statistical analysis

IBM SPSS v26.0 software (IBM SPSS Inc., Chicago, IL, USA) was used. The data of UWSF and SWSF were non-normally distributed continuous variables, so they were presented in medians and quartiles. Categorical variable data were presented as frequencies and percentages (%). Differences between categorical variables among different groups were assessed using chi-square test or Fisher’s exact test. The Mann–Whitney test was employed for comparing differences between two independent samples of non-normally distributed continuous variables. Pearson correlation coefficient was utilized to examine linear relationships between variables. Intraclass correlation coefficient (ICC) was used to assess the relationship between UWSF_5 min and UWSF_10 min. Receiver operating characteristic (ROC) curve and area under the curve (AUC) analysis were employed to evaluate the ability of UWSF to predict and reflect the reduced SG secretion function in SjS patients. All tests were two-tailed, and *P* < 0.05 were considered statistically significant.

## Results

### Clinical characteristics of subjects with suspected SjS

A total of 811 subjects with suspected SjS were recruited. Twenty-seven subjects were excluded due to the coexistence of SjS with other CTDs. Fifty subjects were excluded as they failed to undergo UWSF or SWSF measurements due to severe gingival bleeding, presence of dentures, or inability to chew at a rate of 60–70 times per minute. Finally, a total of 734 subjects with suspected SjS were included for further analysis, with a mean age of 44.0 ± 11.0 years and predominantly female representation (95.4%, *n* = 700/734).

**Fig. 1:**
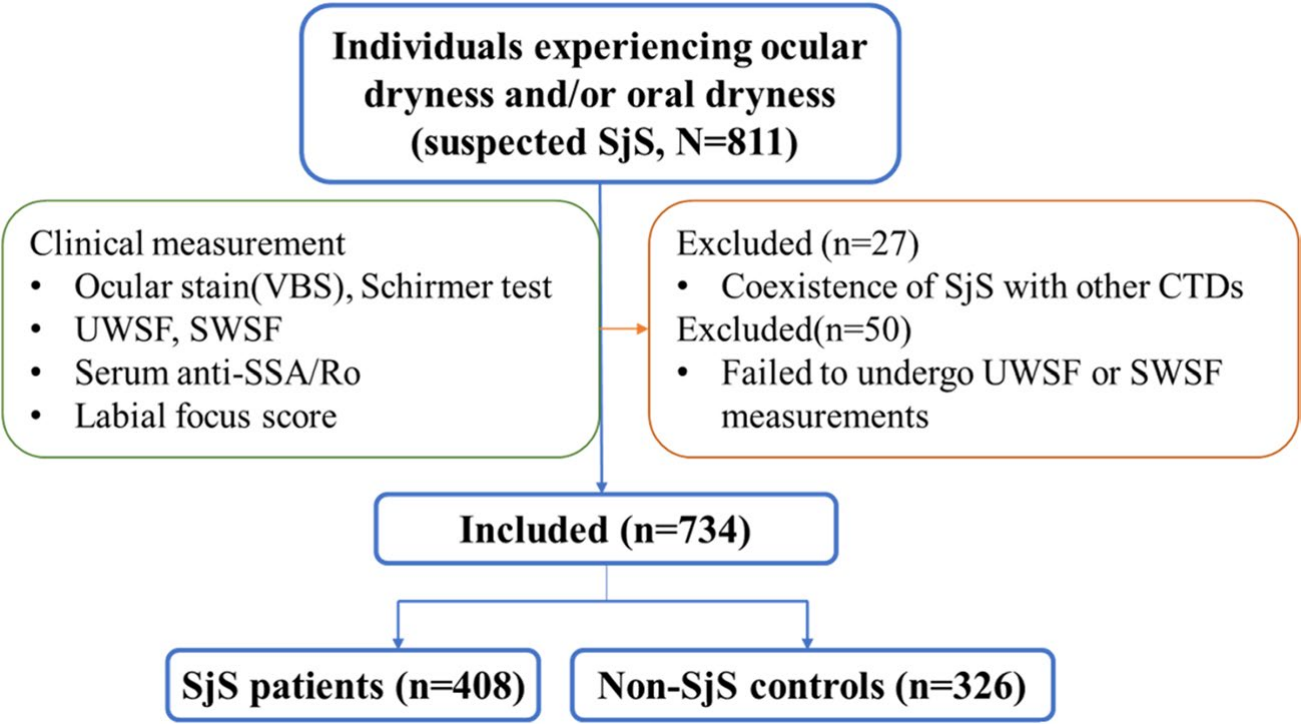
The process of including and excluding participants for the study cohort. A total of 811 individuals suspected to have Sjögren’s syndrome (SjS) were recruited for the study. Among them, 734 participants met the inclusion criteria and did not fulfill the exclusion criteria. They were further divided into two groups: SjS patients (*n* = 408) and non-SjS controls (*n* = 326)

The participants were categorized into two groups: SjS patients (*n* = 408) and non-SjS controls (*n* = 326) (Fig. 1). The mean age of SjS patients was 46.0 ± 11.0 years, with a majority of 95.1% (388/408) being female, which was matched in terms of age and sex to the non-SjS controls (both *P* > 0.05, Table 1).

**Table 1:** Clinical characteristics of patients with Sjögren’s syndrome (SjS) versus non-SjS controls

Characteristics	SjS patients (*n* = 408)	Non-SjS controls (*n* = 326)	*P*
Demographic			
Age, years, mean(SD)	46.0 (11.0)	42.0 (12.0)	0.211
Female, *n* (%)	388 (95.1%)	312 (95.7%)	0.728
Ocular signs			
Schirmer’s test ≤ 5 mm/5 min, *n* (%)	305 (74.7%)	119 (36.5%)	< 0.001
VBS ≥ 4, *n* (%)	220 (53.9%)	60 (18.4%)	< 0.001
SG assessments			
Labial SG focus score ≥ 1, *n* (%)	137 (33.6%)	4 (1.2%)	< 0.001
UWSF_5 min ≤ 0.1 mL/min, *n* (%)	167 (40.9%)	18 (5.5%)	< 0.001
UWSF_10 min ≤ 0.1 mL/min, *n* (%)	154 (37.5%)	8 (2.5%)	< 0.001
Immunological assessments			
Positive Anti-ANA, *n* (%)	385 (94.4%)	178 (54.6%)	< 0.001
Positive Anti-SSA/Ro, *n* (%)	380 (93.1%)	99 (30.4%)	< 0.001
Positive Anti-SSB/La, *n* (%)	149 (36.5%)	29 (8.9%)	< 0.001
Hyperglobulinemia, *n* (%)	194 (47.5%)	63 (19.3%)	< 0.001
Extra-glandular involvements			
Neutropenia, *n* (%)	10 (2.6%)	7 (2.1%)	0.811
Lymphopenia, *n* (%)	62 (15.2%)	25 (7.7%)	0.002
Thrombopenia, *n* (%)	19 (4.7%)	20 (6.1%)	0.408
Albuminuria, *n* (%)	5 (1.2%)	2 (0.6%)	0.471
Interstitial pneumonia, *n* (%)	7 (1.7%)	4 (1.2%)	0.763
Pleural effusion, *n* (%)	2 (0.5%)	2 (0.6%)	1.000
Pericardial effusion, n (%)	1 (0.2%)	0 (0%)	1.000

*VBS* Van Bijsterveld score, *SG* salivary gland, *UWSF* unstimulated whole saliva flow, *ANA* antinuclear antibodies

### The UWSF testing time can be shorten from 10 to 5 min for subjects with suspected SjS

To establish a definitive diagnosis of SjS in clinical practice, it is essential to conduct UWSF measurement in individuals suspected of having SjS. The diagnostic value for SjS was similar between UWSF_10 min [*AUC* 0.743 (95%*CI* 0.708–0.778),* P* < 0.001] and UWSF_5 min [*AUC* 0.735 (95%*CI* 0.699–0.770),* P* < 0.001].

**Fig. 2:**
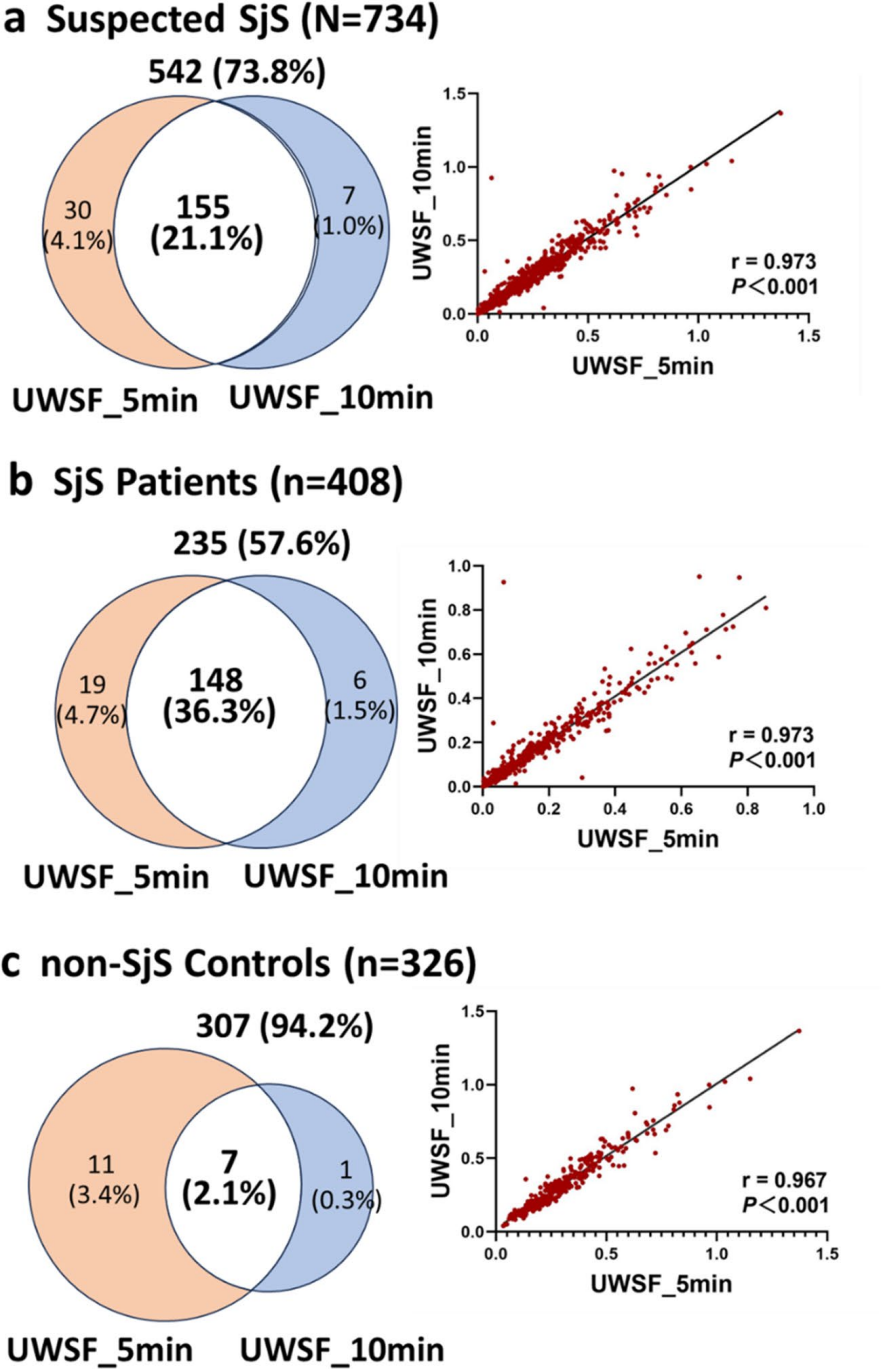
The consistency analysis was conducted between UWSF_5 min and UWSF_10 min in suspected Sjögren's syndrome (SjS) (**a**), confirmed SjS patients (**b**), and non-SjS controls (**c**). The right panels were Venn diagrams which were used to analyze the shared and unique positive and negative efficacy between different test times. The overlapping area of two circles in the Wayne diagram indicates a positive result for both UWSF_10 min and UWSF_5 min, while the area outside the circles signifies a negative result for both UWSF_10 min and UWSF_5 min. The left pink section indicates a positive UWSF_5 min but a negative UWSF_10 min, while the right blue section signifies a positive UWSF_10 min but a negative UWSF_5 min. The right panels displayed scatter diagrams illustrating the linear relationship between UWSF_5 min and UWSF_10 min, along with the corresponding Pearson correlation coefficient (*r*)

In order to optimize efficiency, we investigated the possibility of shortening the UWSF measurement time from 10 to 5 min through a pair-matched comparison involving 734 subjects with suspected SjS. Only 7 subjects (1.0%) exhibited positive UWSF_10 min but negative UWSF_5 min, and 30 subjects (4.1%) had negative UWSF_10 min but positive UWSF_5 min. As a result, 697 subjects (95.0%) showed consistent positive or negative results for both UWSF_5 min and UWSF_10 min (Fig. 2a). The agreement between UWSF_5 min and UWSF_10 min was remarkably high among subjects with suspected SjS (*ICC* 0.970, *Pi* < 0.001; *r* 0.973, *P* < 0.001), SjS patients (*ICC* 0.971, *P* < 0.001; *r* 0.973, *P* < 0.001), as well as non-SjS controls (*ICC* 0.962, *P* < 0.001; *r* 0.967, *P* < 0.001) (Fig. 2). When UWSF_5 min was substituted for UWSF_10 min in 734 subjects with suspected SjS, the positive predictive value (PPV) was determined to be 83.8% (155/185), while the negative predictive value (NPV) reached 98.7% (542/549), resulting in an accuracy rate of 95.0% (697/734).

### Challenges in SWSF measurement all over China

**Fig. 3:**
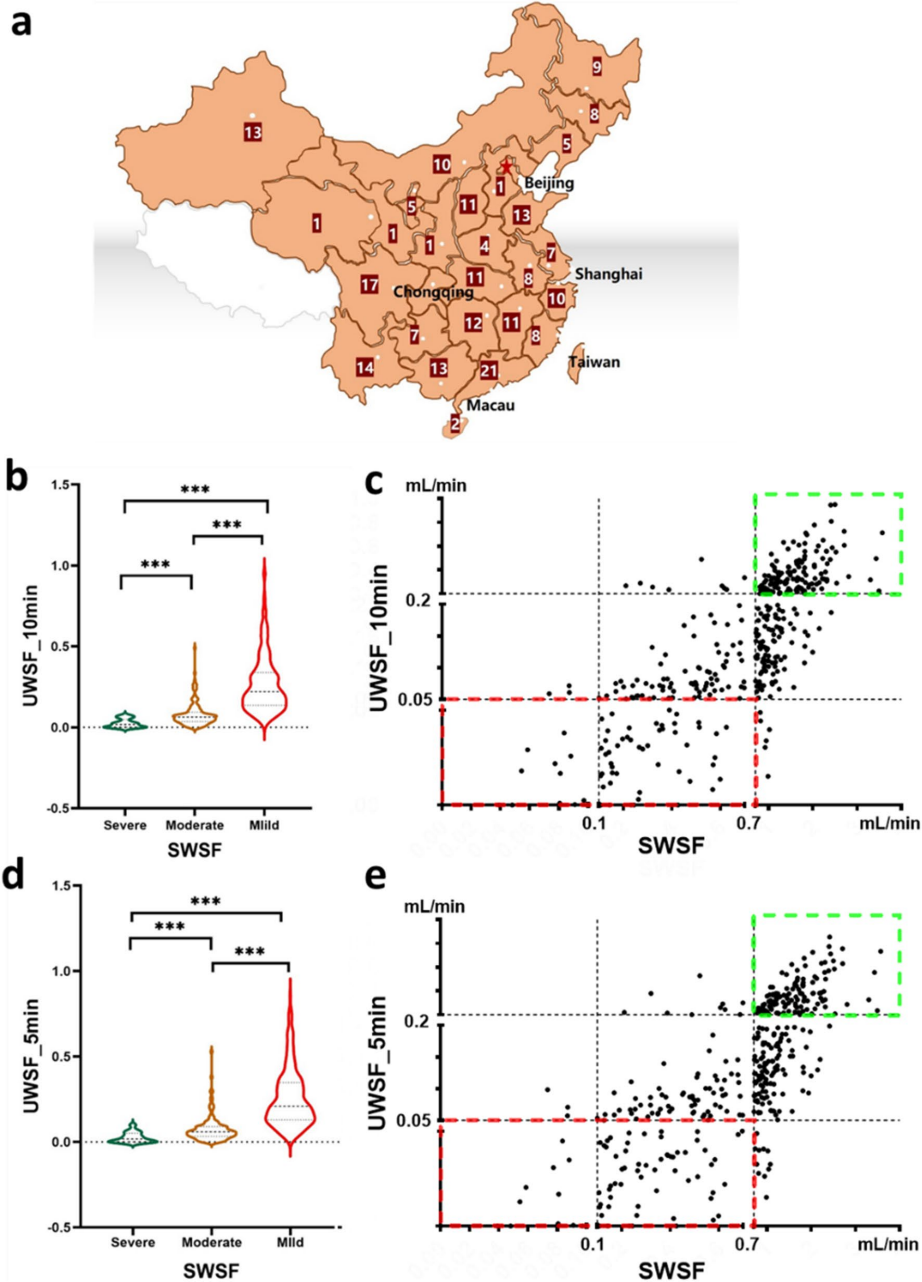
The correlation between UWSF and SWSF results in Sjögren's syndrome (SjS). **a** An online questionnaire survey was conducted among rheumatologists from 660 hospitals in 225 cities and 32 provinces. The digits indicated the number of cities in each province. **b**, **d** UWSF measurements were taken at 10 min (UWSF_10 min, **b**) and at 5 min (UWSF_5 min, **d**). The UWSF results were compared among SjS patients with mild, moderate and severe SG secretory hypofunction. **c**, **e** The scatter diagrams indicated the correlation between SWSF values and UWSF_10 min (**c**) or UWSF_5 min (**e**) results. The green box indicates the correlation between a threshold of UWSF > 0.2 mL/min and SWSF > 0.7 mL/min (indicating mild secretory hypofunction) and the red box represents the correlation between a threshold of UWSF < 0.05 mL/min and SWSF ≤ 0.7 mL/min (indicating moderate to severe secretory hypofunction). ***P < 0.001

When a patient is diagnosed SjS, SWSF measurement is recommended for the stratification of SG secretory function prior to oral dryness treatment [12]. To investigate the prevalence of SWSF measurement across China, an online questionnaire survey was conducted among rheumatologists from 660 hospitals in 225 cities and 32 provinces between May and July 2023 (Fig. 3a). Only 15.3% (101/660) of Chinese hospitals offered SWSF measurement services. Furthermore, a significant proportion of hospitals (96.1%) reported various challenges associated with conducting SWSF measurements, including time constraints (26.8%), inadequate staffing levels (44.2%), and lack of awareness regarding this test (29.2%).

### *SWSF measurement is necessary for SjS patients with UWSF 0.1* ~ *0.2 mL/min*

Despite the 2019 treatment recommendation for SjS [12], we measured SWSF in all 408 SjS patients, even though it was not recommended for those with UWSF ≥ 0.1 mL/min. We unexpectedly found that 21.2% (22/104) of SjS patients with UWSF_10 min 0.1 ~ 0.2 mL/min had moderate secretory hypofunction (SWSF 0.1 ~ 0.7 mL/min). This challenges the 2019 treatment recommendation for SjS and suggests that SjS patients with UWSF_10 min 0.1 ~ 0.2 mL/min also need SWSF measurement.

### UWSF results can stratify SG secretory function in over half of SjS patients

Out of 408 SjS patients, SG secretory hypofunction was classified as mild (SWSF > 0.7 mL/min, *n* = 253, 62.0%), moderate (SWSF 0.1 ~ 0.7 mL/min, *n* = 138, 33.8%), and severe (SWSF < 0.1 mL/min, *n* = 17, 4.2%). The UWSF_10 min level in SjS patients with severe SG secretory hypofunction exhibited a significantly lower value compared to those with moderate SG secretory hypofunction (0.018 ± 0.03 vs. 0.06 ± 0.03, mL/min, *P* < 0.001), or those with mild SG secretory hypofunction (0.018 ± 0.03 vs. 22 ± 0.12, mL/min, *P* < 0.001; Fig. 3b). The cut-off values of UWSF_10 min results were investigated to classify SG secretory function in SjS patients. The relationship between SWSF values and UWSF results was demonstrated through the use of scatter diagrams (Fig. 3c). The green box indicates the correlation between a threshold of UWSF > 0.2 mL/min and SWSF > 0.7 mL/min and the red box represents the correlation between a threshold of UWSF < 0.05 mL/min and SWSF ≤ 0.7 mL/min.

When employing a threshold of UWSF_10 min > 0.2 mL/min (36.8%, 150/408) instead of SWSF > 0.7 mL/min (indicating mild secretory hypofunction), the specificity and PPV were found to be 94.2% (146/155) and 94.0% (141/150), respectively. Conversely, when using a threshold of UWSF_10 min < 0.05 mL/min (16.9%, 69/408) instead of SWSF ≤ 0.7 mL/min (indicating moderate to severe secretory hypofunction), the specificity was remarkably high at 97.6% (247/253), accompanied by a high PPV of 91.3% (63/69). As UWSF testing time can be reduced from 10 to 5 min for diagnosing SjS, similar findings showed UWSF_5 min could classify SG secretory function as accurately as UWSF_10 min (Fig. 3d-e).

## Discussion

The present study presents three significant findings. Firstly, we recruited a diagnostic cohort comprising 734 patients with clinical suspicion of SjS. Our results demonstrate a remarkably high concordance between UWSF measurements taken at 10 min and those taken at 5 min. Moreover, reducing the testing time for UWSF to 5 min resulted in a high PPV of 83.8% and an exceptionally high NPV of 98.7%. These findings provide compelling evidence supporting the possibility of shortening the testing time for UWSF from 10 to 5 min. Secondly, we conducted SWSF measurement to stratify xerostomia severity in a cohort of 408 patients diagnosed with established SjS, unexpectedly revealing that moderate secretory hypofunction was present in 21.2% of patients with UWSF 0.1 ~ 0.2 mL/min. This finding challenges the 2019 treatment recommendation for SjS, which does not require SWSF measurement in SjS patients with UWSF ≥ 0.1 mL/min. Lastly, based on exceptionally high specificity and PPV values obtained using a threshold of UWSF measurement greater than 0.2 mL/min to indicate mild secretory hypofunction or less than 0.05 mL/min to indicate moderate to severe secretory hypofunction, we propose that it may be feasible to consider utilizing UWSF instead of SWSF test for objective classification of SG secretory function in over half of SjS patients.

As a straightforward and pragmatic screening tool, UWSF can be employed by clinicians for initial measurement of individuals with xerostomia suspected of having SjS, thereby facilitating the decision-making process regarding the necessity for further labial gland biopsy and other relevant diagnostic tests. The prevailing belief is that the longer it takes to collect a sample of saliva, the more reliable it will be [17]. Thus the classical 2002 AECG classification initially set at 15 min [7], the testing time for UWSF has proven to be time-consuming and poorly tolerated by patients in routine clinical practice, leading to a gradual reduction in duration. To date, there remains no consensus regarding the optimal timing for UWSF collection, and the recent 2016 ACR/EULAR criteria have eliminated any restrictions on the collection time [6]. Currently, in most clinical scenarios, a recommended duration of 10 min is advised for UWSF collection. This study provides theoretical evidence supporting a reduction in UWSF testing time to 5 min in patients with suspected SjS, while maintaining equal diagnostic value for SjS. Reducing the collection time of UWSF is advantageous for the practical advancement of clinical work and can enhance patient acceptance and cooperation. Therefore, it is recommended to promote the utilization of the UWSF_5 min in clinical practice to alleviate the inconvenience associated with UWSF measurement.

Influenced by environmental and personal stressors, the patient’s subjective experience of xerostomia may not always align with objective measurements of SG secretory function [18]. Rather than relying solely on subjective perceptions, the EULAR-Sjögren’s Syndrome Task Force Group recommends assessing baseline secretory function through quantifying SWSF in SjS with UWSF < 0.1 mL/min [12]. However, a higher proportion of SjS patients present with UWSF ≥ 0.1 mL/min, as evidenced by the fact that over 59% in our study and approximately 45 ~ 65% reported in previous literature [19, 20]. This suggests that there is a higher proportion of SjS patients presenting with UWSF ≥ 0.1 mL/min. So far, there have been no studies providing the results of SWSF in SjS patients with UWSF not less than 0.1 mL/min. Surprisingly, a significant proportion (up to 21.2%) of SjS patients with UWSF between 0.1 and 0.2 mL/min in this study also exhibited moderate secretory hypofunction, highlighting the necessity for measuring SWSF in SjS patients with UWSF between 0.1 and 0.2 mL/min.

**Fig. 4:**
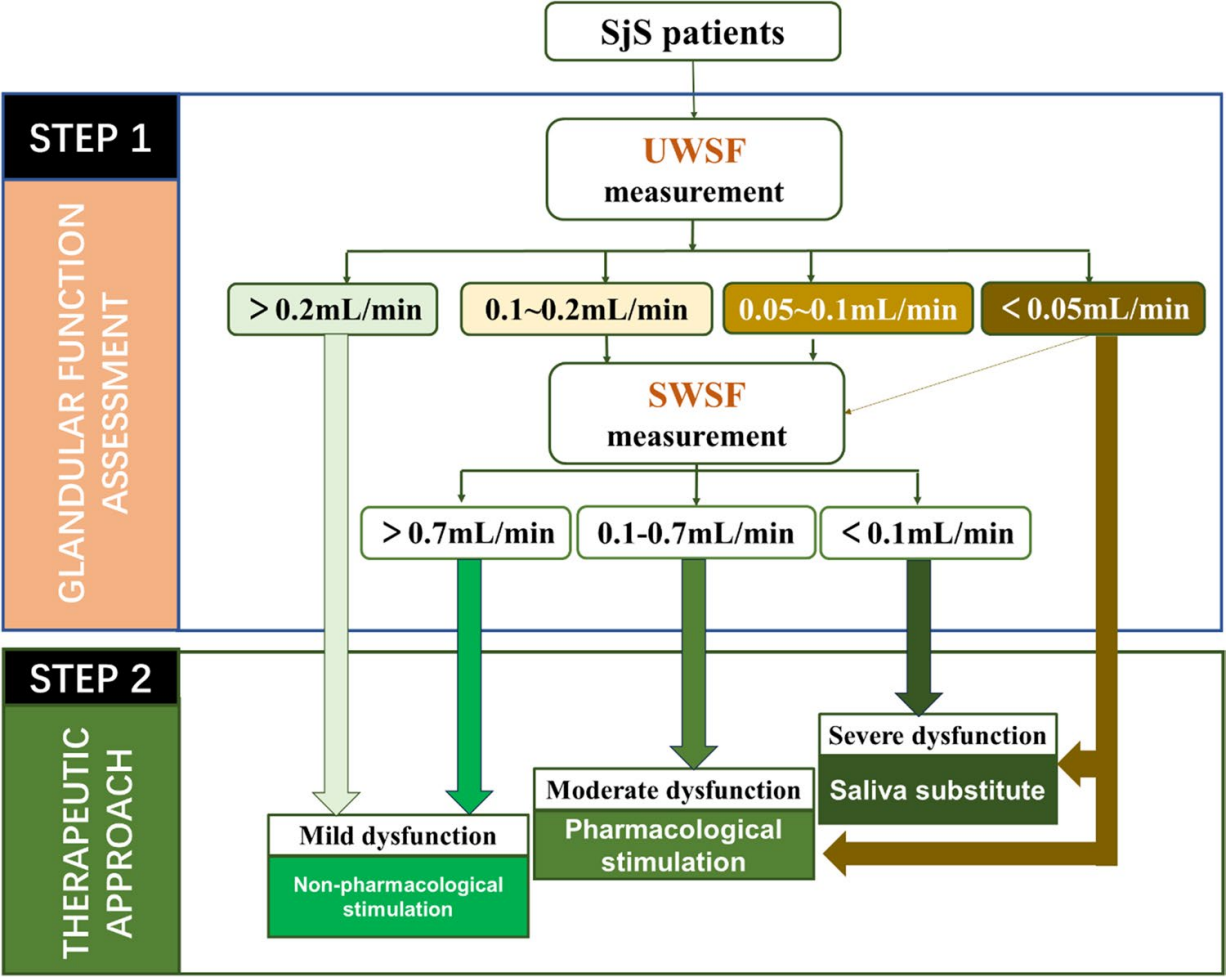
A revised algorithm for assessing glandular function in patients with Sjögren’s syndrome (SjS). UWSF results above a threshold of 0.2 mL/min indicate mild secretory hypofunction, while results below 0.05 mL/min indicate moderate to severe secretory hypofunction. For SjS patients with UWSF ranging from 0.05 to 0.2 mL/min, measuring SWSF is still recommended

Significantly, the current study has highlighted the challenges in assessing SWSF across China. Only 15.3% of Chinese hospitals offer SWSF measurement services, according to an online questionnaire survey of rheumatologists from 660 hospitals in 225 cities. The findings revealed time constraints, inadequate staffing levels, and a lack of awareness regarding this test, which impede the implementation of SWSF measurements. Additionally, 50 participants were excluded prior to the study due to severe gingival bleeding, presence of dentures, and inability to achieve the specified chewing rate. These findings further substantiate the notion that the administration of the SWSF test entails a relatively intricate process in routine clinical practice, rendering it unsuitable for implementation across all clinical settings. To minimize the necessity for SWSF testing, we have developed a revised algorithm for assessing glandular function in SjS patients. This algorithm is based on the assessment of glandular function and therapeutic approach in SjS patients presenting with oral dryness, as outlined in the 2019 treatment recommendation [12]. This procedure can be based on either UWSF_10 min or UWSF_5 min, which were measured during the diagnosis (Fig. 4). Our study suggests that UWSF results above a threshold of 0.2 mL/min can indicate mild secretory hypofunction in SjS patients, while results below 0.0 5 mL/min can indicate moderate to severe secretory hypofunction in this patient population, affecting over half of the patients. These findings have the potential to improve current treatment recommendations regarding which SjS patients should undergo SWSF testing.

We observed that the thresholds for UWSF results were defined differently depending on the specific purpose. In various classification criteria for diagnosing SjS, a positive UWSF was defined as ≤ 0.1 mL/min [6, 7]. On the other hand, the 2019 treatment recommendation for SjS suggested measuring SWSF for those with UWSF < 0.1 mL/min [12]. However, there is currently no explanation provided for this recommendation, and it is speculated that the intention behind it is to better focus on patients who are more eager to undergo SWSF measurement. Moreover, prospective cohort studies are imperative to furnish compelling evidence supporting the practical application of our findings in therapeutic decision-making. Nonetheless, this endeavor sets forth a lucid and innovative trajectory for further exploration in this field. The validation of our findings in participant groups from only 2 study centers, as well as regional and district limitations, may impact the credibility of our conclusions. We encountered some limitations with the sample size in the present study, which unfortunately prevented us from conducting more detailed subgroup analyses using ESSDAI, ESSPRI, or anti-SSA to explore the impact of these indexes on salivary gland function in SjS patients. We apologize for any inconvenience this may have caused and hope to address this issue in future research. To strengthen the evidence, further validation of our findings in more clinical centers and a broader population of individuals with suspected and confirmed SjS is needed.

## Conclusions

This study offers compelling evidence that supports the possibility of reducing the testing time for UWSF to 5 min; and the SWSF test may be skipped for SjS patients with USWF > 0.2 mL/min, indicating mild secretory hypofunction, or < 0.05 mL/min, indicating moderate to severe secretory hypofunction.

## Data Availability

A part of datasets generated and/or analyzed during the current study are not publicly available due patient privacy but are available from the corresponding author on reasonable request.
